# Impact of climate on water status, growth, yield, and phenology of coffee (*Coffea arabica*) plants in the central region of the state of Veracruz, Mexico

**DOI:** 10.1371/journal.pone.0319670

**Published:** 2025-04-21

**Authors:** Paulo César Parada-Molina, Carlos Roberto Cerdán-Cabrera, Juan Cervantes-Pérez, Víctor L. Barradas, Gustavo Celestino Ortiz-Ceballos

**Affiliations:** 1 Facultad de Ciencias Agrícolas, Universidad Veracruzana, Xalapa, Veracruz, México; 2 Facultad de Instrumentación Electrónica, Universidad Veracruzana, Xalapa, Veracruz, México; 3 Instituto de Ecología, Universidad Nacional Autónoma de México, Cd., México; Institute for Biological Research, University of Belgrade, SERBIA

## Abstract

Coffee (*Coffea arabica*) is one of the most widely traded and most consumed agro-products worldwide. Its production is concentrated in tropical regions, and its consumption, in northern countries. Climate variability influences coffee yield and quality, and the distribution of wet and dry periods is closely related to its phenological phases. Recently, the vulnerability of coffee producing regions to changes in climate patterns has been demonstrated. Therefore, this study evaluated the effect of climatic variables on the water status, vegetative growth, yield, and phenology of coffee plants. The research was carried out in a coffee agroecosystem (Garnica variety) located in the central region of the state of Veracruz, Mexico (19.51998^∘^ N and 96.94339^∘^ W; 1320 masl). For three years, the phenology of coffee plants was monitored; plant growth (height, number of leaves) and cherry yield were measured each month during three productive periods. Microclimatic variables (temperature, precipitation, relative air humidity, solar radiation, and wind direction) and water-balance variables (infiltration, rainfall interception, transpiration, soil water storage, crop evapotranspiration [ETo], and reference evapotranspiration [ETc]) were also monitored. The water status of the plants was evaluated based on their water demand, determined as the ETc/ETo ratio. The relationship of microclimatic variables with water status, plant growth, and plant yield was measured by performing correlation statistical tests (Pearson; α=0.05), principal component analyses (PCA), and simple and multiple linear regressions. The results show that the highest water consumption occurred during the flowering (ETc/ETo=1.12), and grain ripening (ETc/ETo=1.15) phenological phases, while the lowest value (ETc/ETo=0.62), indicative of water deficit, was observed at harvest for the period 2018–2019. Precipitation (P) and rainfall infiltration (I) are the variables with the greatest influence on vegetative growth (*r*^2^>0.70). A relationship was observed between yield and water and microclimatic variables. However, simple and multiple linear regressions, including PCA, explain less than 45% (*p* < 0.05) of the variability of yield data. This variability is mainly described by water conditions related to soil water storage (S) and thermal conditions, particularly the minimum temperature (Tmin). Our findings suggest that the water demand of coffee plants changes significantly with the phenological phases of the crop; therefore, changes in the cyclical patterns of climate variation could cause a water deficit in coffee plants, limiting their development, yield, and quality.

## Introduction

Coffee is one of the most important agro-products worldwide; *Coffea arabica* is the most widely distributed and consumed species [1]. *C. arabica* has been classified as sensitive to changes in climate patterns [2]. Climate variability drives the dynamics of its production, modulating coffee production cycles [3–5]; this makes coffee vulnerable because its phenological development and vegetative growth are closely related to the temporal distribution of wet and dry season, air temperature; and photoperiods [6, 7].

Drastic variations in the amount, intensity, and frequency of precipitation events influence the temporal availability of water for crop development [8]. The different phenological phases of coffee plants have different water needs, so changes in water availability can affect their development and yield. For example; the “spring” water deficit is necessary to stimulate flowering [9]; however, a persistent drought during this season can disrupt it and trigger defoliation as a defense mechanism [10–12].

Water availability during the fruit growth and filling phases is a critical factor that directly impacts yield [13, 14]; fruits reach larger sizes when there are sufficient soil water reserves [13]. In this regard, Gonzales *et al*. [15]; reported low yields due to a decrease in fruit growth and fruit filling caused by water stress. Do Nascimento [16] reported that, in a plot of organic coffee, the absence of water stress reduced the amount of dry and vain fruits and increased the amount of green fruits and the yield of the plants. On the other hand, De Oliveira [17] studied water deficits in arabica coffee plants under controlled conditions in localities of the state of Minas Gerais, Brazil. In this study, in high yield cycles, the water deficit affected the reproductive stage of coffee plants, to a greater extent, while in low yield cycles, it primarily affected the vegetative stage. Finally, Ronchi [18] confirmed that water deficit during the pre-flowering stage of arabica coffee plants improves flowering uniformity.

A parameter that indicates crop water demand is the ratio between crop evapotranspiration and reference evapotranspiration (ETc/ETo) [19]. This parameter expresses the capacity of plants to extract water from the soil and provides insight into the water status of crops. Values close to 1 reflect higher water consumption. For *C. arabica* varieties under controlled conditions, mean values between 0.86 and 0.88 have been reported for the productive cycle [20, 21]. Additionally, values vary for each phenological phase of the crop, with the flowering and ripening phases showing values close to one, indicating that these phases have higher water needs [20, 22].

From the above, changes in climate patterns are expected to impact the development of coffee cultivation; however, such impacts of climate change are not reflected similarly in the different coffee growing regions of the world. According to Wagner [23], in the equatorial region of Tanzania, at altitudes between 1000 m and 1800 masl, longer dry periods adversely impact *C. arabica* production and yield; meanwhile, in tropical regions of Brazil (13 ^∘^C to 20 ^∘^C), no adverse effects on crop production are expected [24]. In the most important coffee-production region in the state of Veracruz, Mexico, there have been temporal variations in the amount and intensity of precipitation, including an longer dry periods, which could induce water deficit stress in coffee plants [25]. Therefore, the objective of this study was to evaluate the impact of microclimatic variables on the water status, growth, yield and phenology of *C. arabica* plants in the central region of the state of Veracruz, Mexico.

## Materials and methods

### Description of the study site and plant material

This study was carried out in the “La Herradura” coffee plantation (19.51998^∘^ N and 96.94339^∘^ W; 1320 masl) which is representative of the coffee plantations in the central region of the state of Veracruz, Mexico. The ranch is owned by cofee producer Ing. Roberto Licona, who provided all the necessary permits and facilities to set up the experiment. According to the National Meteorological System of Mexico (SMN, for its acronym in Spanish) (2020), the meteorological station closest to the study site is the Briones station (located at 500 m and 1349 masl), with a humid warm climate Cw (fm) (i’) w” [26], annual precipitation of 1699.3 mm; mean annual temperature of 18 ^∘^C, and maximum and minimum temperatures of 25 ^∘^C and 11 ^∘^C, respectively.

“La Herradura” plantation comprises an area of 11 ha under agroforestry management; 80% of the coffee plants are *C. arabica*, Garnica variety, which is a cross between the Mundo Novo and Caturra varieties; the remaining 20% is composed of other commercial varieties. The Garnica variety is considered good cup quality, with a medium yield but susceptible to coffee rust. The planting density is 1600 plants ha-1, which is low compared to the recommended density for this variety. Specific agricultural practices are used, such as transplanting the plants to 20 cm radius and 40 cm deep planting beds where the soil is replaced by compost, placing dolomite rocks near the plants to control soil acidity, and maintaining a cover of herbs with creeping growth to preserve soil humidity. The tree component consists of pink cedar (*Acrocarpus fraxinifolius*) at a density of 278 trees ha-1. Other identified tree species that are randomy distributed around the plot, include chalahuites (*Inga sp.*), gravilea (*Grevillea robusta*), piocho (*Melia azedarach*), neem tree (*Azadirachta indica*), Indian tulip (*Spathodea campanulata*), ixpepel (*Trema micrantha*), and some fruit trees. Additionally, there are fragments of preserved cloud forest three kilometers away.

### Delimitation of the sampling unit

A 40 m x 60 m plot was delimited within the plantation. The coffee plants were approximately 15 years old and were subjected to a rejuvenation pruning at 10 years of age. Within the plot, 30 coffee plants in a productive state were selected at random. the water status, growth, number of leaves, and yield of each selected coffee plant were recorded from may 2017 to february 2020 (34 months), corresponding to the duration of the phenological phases. This period included 3 annual harvests.

### Monitoring of microclimatic variables

A Davis Vantage Pro2 TM automated weather station was installed inside the plot. The sensors were installed 2 m above coffee plants to record temperature (T, ^∘^C), relative humidity (RH, %), solar radiation ($W/{\text{m}}^{2}$), wind direction, and speed (m/s) every 15 minutes. Precipitation (P) was recorded with a pluviometer (HOBO-Onset) installed at an open site within the plantation. In addition, the precipitation data was compared with average precipitation data from the Briones station of the National Meteorological Service (period 1981–2010), the station closest to the sampling site.

### Yield, growth, and duration of the phenological phases

In each of the 30 selected coffee plants, yield and growth were estimated during three productive periods (May 2017 to February 2020). To measure coffee yield, one productive plagiotropic branch per plant facing north was selected and labeled; ripe fruits were harvested, and their weight (g/plant) and percentage of ripe and empty fruits were recorded. These variables were estimated during the harvest season and added to estimate annual data per plant. Vegetative growth was evaluated monthly by measuring plant height from the ground to the highest growing point and counting the number of leaves per plant. Each plagiotropic branches was monitored weekly to identify the Julian day of the beginning of the phenological phases of the plants: flowering (F), fruit growth and filling (G), ripening (R), and harvest (H). The duration (in weeks) of the phenological phases ($f_{week}$) of each coffee plant was determined using the “Extended BBCH Scale” [27]. To estimate the flowering period of the missing productive cycle (February, March and April 2017), the plantation producer was consulted to determine the beginning of this phenological phase.

### Water status

Water status was determined by estimating the water demand of coffee plants from the ratio between crop evapotranspiration and reference evapotranspiration (ETc/ETo) [19]. ETc was determined as the sum of transpiration (Tr) and rainfall interception (IN). *Tr* was calculated as the residual of the water balance equation using the mass balance method [28]:


Tr=P−(IN+Q+I+S)

where Tr is coffee and tree transpiration; P is precipitation; IN is rainfall interception; Q is surface runoff; I is water infiltration in the upper 50 cm of soil; and S is the variation in soil water content. All values are expressed in mm. Surface runoff was excluded because the study area was small, with a slope of less than 5%, and because of leaf litter from the tree cover. The reference evapotranspiration (ETo) was estimated using the FAO Penman-Monteith equation [29].

**Fig 1:**
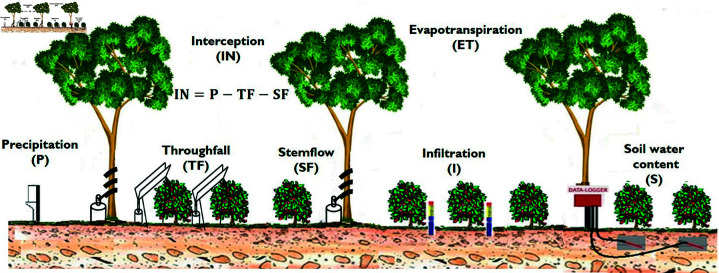
Diagram of the water balance variables evaluated in the four subplots.

The components of the water balance were evaluated in four subplots of 28.27*m*^2^ and 3 m radius, with the trunk of a shade tree as the center; we selected 2 coffee plants located at 1 m (Â±0.20 m) and 2 m (Â±0.20 m) away from the tree trunk. The water variables were recorded at the same distances (Fig 1).

Interception (IN) was estimated as the difference between incident precipitation and transcolation (TF) and cortical flow (SF). In shade trees and coffee plants, TF was measured using “V” shaped galvanized sheeting gutters with an opening angle of 60^∘^ and placed at an inclination of 30^∘^. The TF of the shade trees was measured with troughs 1.2 m long by 0.20 m wide ($area=0.24m^{2}$) at 1 m above the ground; in coffee plants, TF was estimated with troughs 0.80 m long by 0.10 m wide ($area=0.08m^{2}$), at 0.40 m above the ground. The SF was measured in four shade trees using 1.5 m long spiral-shaped PVC plastic troughs fixed on the trunk of the trees between 0.5 m and 1.2 m from the trunk base. In coffee plants, the SF was estimated only from May 2019 to February 2020 using funnel-shaped PVC troughs. All troughs were connected to 20 L and 4 L collector containers with lids.

TF in the water sheet (mm) was determined by dividing the number of liters by the catchment area of the gutters; to estimate SF (mm), the volume measured per coffee tree and plant was multiplied by the planting density (278 trees ha-1 and 1600 plants ha-1, respectively) and divided by 10000*m*^2^. Both fluxes (TF and SF) were measured manually every 3 days using a graduated container accurate to 1 mL.

Infiltration (I) was calculated from the basic infiltration measured in the field, according to Schosinsky [30]. Soil water content ($m^{3}/m^{3}$) was measured with 5TM probes, connected to an EM50 datalogger (Decagon Devices Inc.) at 0.3 m and 0.5 m depth. Total soil water content (S) was calculated as the cumulative sum of volumetric water content in the layers (in mm) multiplied by the thickness of each layer.

### Statistical analysis

ANOVA tests were performed to determine differences in the duration (weeks) of the phenological phases between productive periods and to identify statistical differences in the behavior of the microclimatic and water variables in each phenological phase by productive period. The variables that showed significant differences were subjected to a Tukey‘s test (*p* < 0.05; NS 95% significance level). The response of coffee plants growth and yield to the water variables monitored at one and two m distance from the trunk of shade trees was evaluated by a Student‘s test; however, since no differences were found between distances, these data were pooled. The relationship between water, microclimatic variables, vegetative growth, and yield of the sampled plants was explored using the Pearson statistical test (Pearson; α=0.05). Additionally, a Principal Component Analysis (PCA) was performed to identify the water and microclimatic variables that contribute the most to explaining yield and vegetative growth. Finally, simple and multiple linear regression models were estimated to measure the relationship between water and microclimatic variables and the yield response variable. In addition, a multiple linear regression model was run in which the indices generated in the principal components one and two derived from the PCA were the explanatory variables. The statistical software used in this study is SPSS [31].

## Results

### Influence of microclimatic variables on coffee plants phenology

The duration (in days) of each of the four phenological phases (flowering, fruit growth and filling, ripening, and harvest) was statistically similar between the productive cycles evaluated. Only the Julian day of the end of the fruit growth and filling phase (G) and its duration, in weeks (${\mathrm{Ll}}_{\mathrm{week}}$) showed significant differences (*p* < 0.02) between productive period, being longer in the second one Table 1.

**Table 1:** Duration of the phenological phases of cofee plants.

Period	Phenological phase	*N*_f_i_		*N*_f_f_		*f*_week_	
		(days)	sd	(days)	sd	(weeks)	sd
First period 2017–2018	Flowering	50.0		113.0		9.0	
	Growth and filling	114.9	10.2	293.3^*a*^	15.8	25.5^*a*^	2.3
	Ripening	293.4	11.2	344.9	11.5	7.4	0.8
	Harvest	344.1	12.7	48.9	8.7	10.0	2.4
Second period 2018–2019	Flowering	59.5	8.4	123.3	9.0	9.1	1.6
	Growth and filling	113.9	10.9	302.8^*b*^	10.4	27.0^*b*^	1.5
	Ripening	303.8	10.4	354.4	11.8	7.2	1.4
	Harvest	351.9	14.7	58.5	8.4	10.2	2.5
Third period 2019–2020	Flowering	49.9	8.4	112.2	10.1	8.9	1.8
	Growth and filling	115.9	10.7	289.0^*a*^	11.9	24.7^*a*^	2.1
	Ripening	294.2	10.6	345.4	11.3	7.3	1.3
	Harvest	348.3	14.4	56.2	8.3	10.4	2.2

*N*_f_i_, Julian day at the beginning of each phase; *N*_f_f_, Julian day at the end of each phase; *f week*, duration of each phase, in weeks.

**Fig 2:**
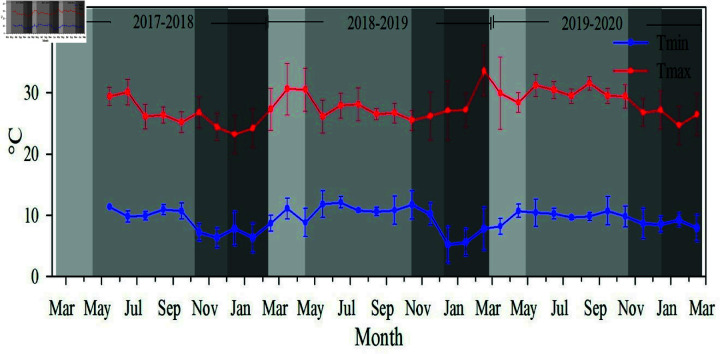
Monthly variation of maximum temperature (red) and minimum (blue) temperature withing the coffee plantation from May 2017 to February 2020. Wires represent the standard deviation of the mean (sd). Shaded areas represent the flowering, fruit growth and filling, ripening, and harvesting phases (from lightest to darkest, respectively).

The differences between daily maximum and minimum temperatures (average by month) were not statistically significant. However, temperatures were slightly lower in the first annual period analyzed (26.3 ^∘^C maximum and 8.9 ^∘^C minimum) and increase in the second period (28.1 ^∘^C and 9.6 ^∘^C) and third periods (28.7 ^∘^C and 9.5 ^∘^C). The highest temperatures occurred in summer and the lowest in winter (Fig 2).

Precipitation during the 2017–2018 productive period was monitored from the beginning of the fruit growth and filling phase until the end of the harvest phase, accounting for cumulative 1828.3 mm. During 2018–2019 and 2019–2020, the cumulative precipitation values were 1583.4 mm and 1681.8 mm, respectively.

**Fig 3:**
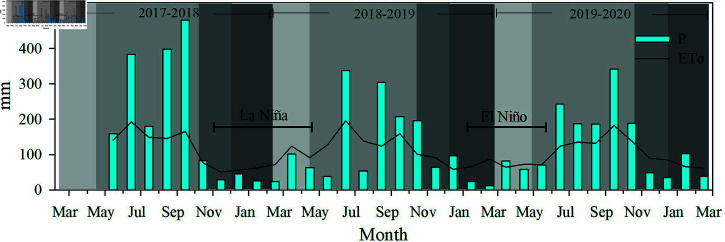
Monthly cumulative precipitation (P) and reference evapotranspiration (ETo) from May 2017 to February 2020. Shaded areas represent the flowering, fruit growth and filling, ripening, and harvesting phases (from lightest to darkest, respectively).

When comparing the cumulative precipitation for each production period with the average precipitation recorded at the Briones Station for the period 1981-2010, we found that the cumulative precipitation during the first productive period (2017–2018) was 20.1% higher than the average precipitation; the second period (2018–2019) was 14% lower, and the third period (2019–2020) was similar to the average precipitation reported for the Briones station. These differences in precipitation observed in the periods 2017–2018 and 2018–2019 are explained by the presence of La NiÃ±a and El NiÃ±o eventos in these periods (Fig 3).

The reference evapotranspiration (ETo) values in each productive period were 1092.9 mm, 1524.3 mm, and 1012.0 mm, respectively, illustrating the seasonal variation typical of the dry and wet seasons (Figure 3).

The behavior of the microclimatic variables in each phenological phases showed significant differences (*p* < 0.05) between the evaluated periods (Table 2). The Tmax reported for each of the phenological phase during the 2019–2020 period was significantly higher than for the 2017–2018 and 2018–2019, except for the ripening phase, for which no significant differences were found. Precipitation also showed significant differences within each phenological phase and between productive periods, except for the flowering phase. These variations in thermal conditions could significantly influence the duration and temporal distribution of the phenological phases, as in the case of fruit growth and filling (Table 2).

**Table 2:** Average behavior of microclimatic variables estimated for each phenological phase in coffee plants (F, flowering; G, fruit growth and filling; R, ripening; H, harvest).

Period	Phase	Tmax		Tmin		P		ET0
		(^∘^C)	sd	(^∘^C)	sd	(mm)	sd	mm
First period 2017–2018	G	27.3^*b*^	2.0	10.0	1.5	1684.1^*c*^	33.7	869.2
	R	23.8^*a*^	0.8	7.1^*a*^	1.1	73.4^*a*^	6.9	106.5^*a*^
	H	24.9^*b*^	2.1	7.6^*b*^	1.2	70.8^*b*^	21.0	117.2^*a*^
Second period 2018–2019	F	29.5^*a*^	1.9	9.5^*a*^	1.4	188.5	25.2	286.9
	G	26.8^*a*^	1.0	11.3	0.6	1198.8^*b*^	46.9	936.3
	R	26.7^*c*^	0.6	7.7^*b*^	1.4	160.3^*c*^	55.0	148.7^*b*^
	H	29.5^*a*^	3.1	6.7^*a*^	1.5	35.8^*a*^	37.7	152.3^*c*^
Third period 2019–2020	F	29.8^*b*^	1.4	9.8^*b*^	1.4	210.2	20.3	208.4
	G	29.8^*c*^	1.6	9.9	0.7	1195.2^*a*^	55.4	799.9
	R	26.2^*b*^	1.3	8.8^*c*^	0.4	138.0^*b*^	28.3	150.6^*c*^
	H	25.6^*b*^	1.2	8.6^*c*^	0.9	141.4^*c*^	18.2	61.41^*b*^

Different letters in the same phenological phase indicate statistical differences (*p* < 0.05) between productive periods.

### Water balances components

Transcolation fluxes (TF), through the canopy of trees and coffee plants, measured at a distance of 1 m and 2 m from shade trees, did not report significant differences (p=0.055); the cortical flux (SF) measured in coffee plants showed a similar bahavior only during the period 2019–2020 at the same distances from shade trees (p=0.052).

The mean TF values ranged from 95% to 97% of precipitation across the tree canopy and 84.7% to 87.1% across the coffee plant canopy. The Mean SF ranged from 0.95% to 1.1% of precipitation through the shade trees and 5.2% through the coffee plants, measured only in the period 2019–2020.

Rainfall interception (IN) by the phenological phase showed significant differences (*p* < 0.01) between productive periods, except during ripening. IN by tree stratum of pink cedar (*A. fraxinifolius*) for all productive periods represented between 1.35% and 3.85%; while the shrub stratum (coffee plants) intercepted between 7.7% and 10.4% of rainfall. The estimated IN by the arboreal stratum was 11.8% (216.1 mm) and 13.9 % (218.6 mm) for the first and second periods, respectively, considering that these did not include the SF of coffee plants; the IN for 2019–2020 was 9.8% (165.2 mm) (Table 3). Therefore, the average amount of water that reached the soil surface was 1612.3 mm (88.2% of precipitation), 1364.8 mm (86.2%) and 1519.6 mm (90.2%) for each period analyzed, respectively.

**Table 3:** Water balance components, reference evapotranspiration (ETo) and ETc/ETo ratio associated with each phenological phase of coffee plants, during three productive periods (2017-2020).

Period	Phase	P	IN	Tr	ETc	S	I	Eto	ETc
		(mm)	(mm)	(mm)	(mm)	(mm)	(mm)	(mm)	ETo
First period 2017–2018	G	1684.1^*c*^	194.7^*c*^	438.2	632.9	134.1^*b*^	917.1^*b*^	869.2	0.73^*c*^
	R	73.4^*a*^	10.7	111.3^*a*^	122.0	-52.6^*a*^	3.9^*a*^	106.5^*a*^	1.15
	H	70.8^*b*^	10.7^*b*^	109.7	120.4	-53.5^*b*^	4.0 a	117.2^*a*^	1.03^*a*^
Second period 2018–2019	F	188.5	27.0^*b*^	261.5	288.4	-124.2	24.3	286.9	1.01^*a*^
	G	1198.8^*b*^	160.7^*b*^	477.1	637.8	123.2^*a*^	437.8^*a*^	936.3	0.68^*a*^
	R	160.3^*c*^	25.3	150.5^*b*^	175.7	-38.7^*c*^	23.2^*b*^	148.7^*b*^	1.18
	H	35.8^*c*^	5.7^*a*^	89.5	95.2	-61.4^*a*^	2.0^*a*^	152.3^*c*^	0.62^*a*^
Third period 2019–2020	F	210.2	22.4^*a*^	235.8	258.2	-84.2	36.2	208.4	1.24^*b*^
	G	1195.2^*a*^	106.5^*a*^	454.4	561.0	211.1^*c*^	423.1^*a*^	799.9	0.70^*b*^
	R	138.0^*b*^	16.1	152.7^*c*^	168.8	-49.2^*b*^	18.4^*b*^	150.6^*c*^	1.12
	H	141.4^*a*^	20.3^*c*^	115.9	136.2	-13.2^*c*^	18.5^*b*^	61.41^*b*^	1.07^*b*^

Columns are as follows: P, precipitation; IN, rainfall interception; Tr, transpiration; ETc, crop evapotranspiration (IN + Tr); S, soil water storage; and I, infiltration. Different letters in the same phase indicate statistically significant differences (*p* < 0.05) between productive periods.

During the period 2017–2018 (excluding the flowering phase), soil water storage (S) in the upper 0.50 m was 1.5% (28 mm) of precipitation. The second period returned negative values (-101.1 mm), while 2019–2020 showed an S value of 3.8% (64.5 mm) of precipitation.

Water storage (*S*) by phenological phase showed significant differences (*p* < 0.01) between productive periods, except for flowering, as did soil water infiltration (I). During the fruit growth and filling phase, soil water storage (S) showed positive values, while the flowering, ripening, and harvest phases returned negative values (Table 3). Infiltration (I) was 50.6% (925 mm) of precipitation in 2017–2018, 30.9% (487.3 mm) in 2018–2019 and 29.45% (496.1 mm) in 2019-20120. The highest I values correspond to fruit growth and filling, followed by flowering (Table 3).

Crop evapotranspiration (ETc=IN+Tr), was 47.9% (875.4 mm) of precipitation in 2017–2018, 75.7% (1197.2 mm) in 2018–2019, and 66.7% (1124.2 mm) in 2019–2020. During the fruit growth and filling phases, rainfall interception (IN) contributed 33.8% of ETc; in the other phases, it contributed less than 12%. ETc for each phenological phase showed higher values for the flowering and fruit growth and filling phases, and lower for the ripening and harvest phases. The reference evapotranspiration (ETo) showed significant differences (*p* < 0.01) in the between productive periods in the ripening and harvest phases, but no differences were observed between the flowering and fruit growth and filling phases (Table 3).

### Water status

The ratio between crop evapotranspiration and reference evapotranspiration (ETc/ETo) indicates the water status of coffee plants [19]. For the ripening and flowering phases (not reported for the period 2017–2018), the (ETc/ETo) values were greater than one in the three periods evaluated, indicating that coffee plants showed higher water consumption during the flowering and ripening phases in the three productive periods evaluated. However, during the period 2018–2019 significantly lower values (*p* < 0.01) were recorded during the flowering (1.01), fruit growth and filling (0.68), and harvest (0.62) phases compared to the other periods (Table 3).

### Vegetative development and yield of coffee plants

**Fig 4:**
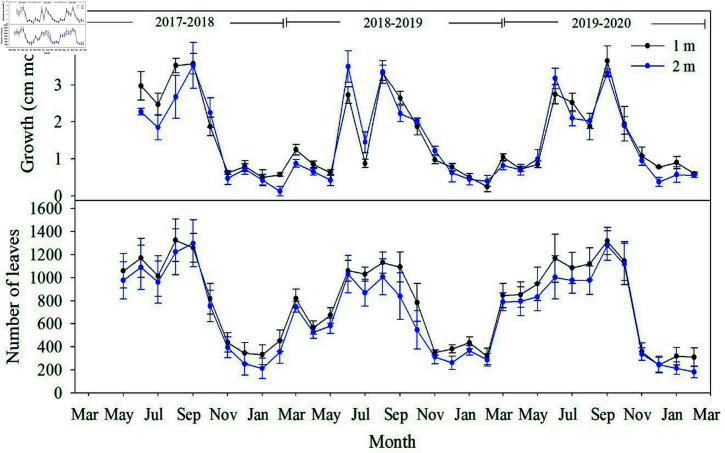
Monthly variation of a) growth and b) number of leaves evaluated in coffee plants located at 1 m and 2 m from the trunk of shade trees during three productive periods from May-2017 to February 2020).

Vegetative growth (height and number of leaves), evaluated at 1 and 2 m distance from the shading trees, did not show significant differences. The growth and number of leaves of the coffee plants sampled presented two peaks during each productive period at the two distances evaluated (Fig 4). For the three annual periods evaluated, the first growth peak (the highest) was in June and the second occurred between August and September. During July and September of the 2018–2019 period, growth was significantly lower (p=0.012 and p=0.048, respectively) compared to the other productive periods; it was also lower in November corresponding to the 2017–2018 period (p=0.026) and during August of the 2019–2020 period (p=0.032). The number of leaves estimated during May presented significant differences for the three productive periods evaluated, reaching its lowest value in 2018–2019 (p=0.048). The mean height growth rates were 1.74, 1.41 and 1.71 cm month-1 for each productive period, respectively.

**Fig 5:**
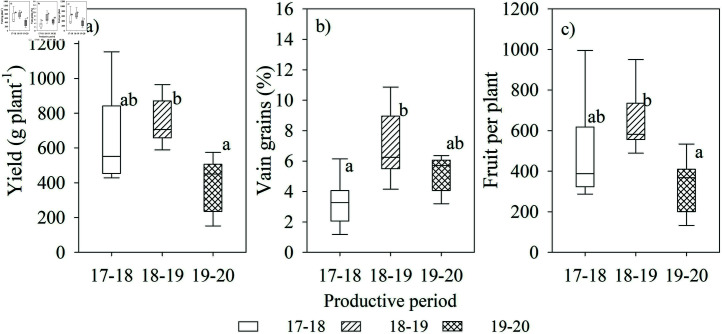
Annual mean of a) yield, b) empty fruits (% of yield), and c) number of empty fruits in coffee plants in three productive periods from May-2017 to February 2020).

The estimated yield between coffee plants sampled at a distance of 1 m and 2 m from the shading trees did not show significant differences, but the differences between production periods were significant (Fig 5).

The average yield per plant was 651.7 g for the period 2017–2018, 755.7 g for 2018–2019, and 373.5 g for 2019–2020, giving an average yield of 593.63 g per plant for the three periods. The high productivity of coffee plants during 2017–2018 and 2018–2019 is related to the higher estimated ETc during those periods (Table 3).

The correlations between vegetative growth variables (growth and number of leaves) and water and temperature variables were estimated (Table 4). The Pearson’s correlation coefficient showed a significant association of growth ($r^{2}=0.85$) and number of leaves ($r^{2}=0.72$) with precipitation (P), infiltration (I) and soil water storage (S).

**Table 4:** Correlation of vegetative growth variables with water variables and temperature. The asterisk marks Significant correlations (*p* < 0.05).

	ETo	P	IN	Tr	ETc	I	S	Tmin	Tmax
Growth	0.72*	0.85*	0.84*	0.26*	0.60*	0.85*	0.74*	0.40*	0.10
No leaves	0.70*	0.72*	0.70*	0.31*	0.58*	0.72*	0.59*	0.46*	0.36*

Columns are as follows: P, precipitation; IN, rainfall interception; Tr, transpiration; ETc, crop evapotranspiration (IN+Tr); S, soil water storage; I, infiltration.

The yield per plant showed a significant positive relationship with Tmin and was negatively related to soil water content. However, according to the simple linear regression models, these variables only explained 22% and 38% of the variation of yield in coffee plants; on the other hand, the multiple linear regression models showed that the variables that best predict yield are Tmax, ETc (40%) and P and ETo (39%), respectively (Table 5).

**Table 5:** Coffee yield models as a function of water, microclimatic and principal component variables using simple and multiple linear regressions.

Model	F	P	*r* ^2^
Yield = -2187.52 + 303.6 Tmin	6.0	0.02	0.22
Yield = 622.80 – 486 S	13.1	0.001	0.38
Yield = -11508.1 + 3.7 P + 4.6 ETo	7.4	0.004	0.39
Yield = 10252.2 – 677.3 Tmax + 9.1 ETc	7.6	0.004	0.40
Yield = 593.6 – 156.2 CP2 – 52.8 CP1	7.1	0.002	0.42

**Fig 6:**
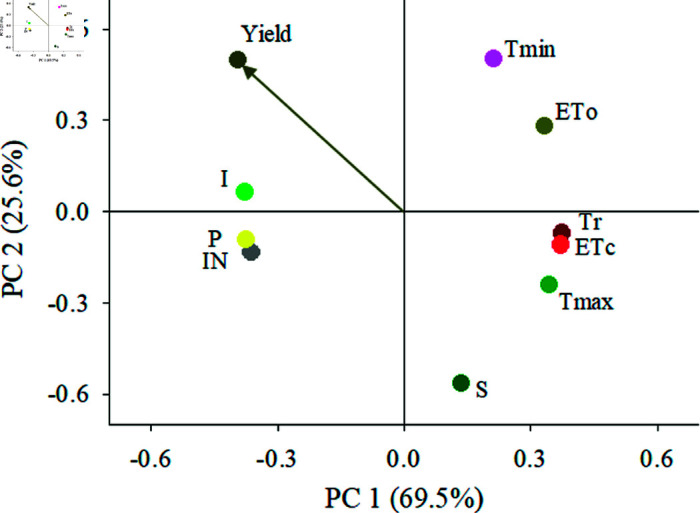
Biplot of Principal Component Analysis (PCA) of annual yield per plant (*g/plant*) as a function of precipitation (P), infiltration (I), rainfall interception (IN), transpiration (Tr), evapotranspiration (ETc), reference evapotranspiration (ETo), soil water storage (S), maximum temperature (Tmax) and minimum temperature (Tmin).

A PCA was carried out to identify the water balance and climate variables that best explain yield, it showed that 95% of the variation produced is explained by components 1 and 2. The variables that contributed the most to PC1 were I, P, IN, Tr, and ETc; while those that contributed the most to PC2 were S and Tmin. Similarly, the PCA showed that I, P, and IN are closely related to each other (Fig 6).

Finally, the multiple linear regression model in which the explanatory variables were the indices generated in the principal components 1 and 2 explained 42% of the variability of yield in coffee plants (Table 5); in addition, it was found that the second component has a greater influence (*p* < 0.002) on yield .

## Discussion

**Influence of microclimatic variables and water status on the phenology of coffee plants** The duration and delimitation of the phenological phases of coffee plants can vary between productive periods in response to changes in temperature, which can cause an overlap between phases. According to Arcila [27] and Barva [32], coffee fruit ripening begins 32 weeks after flowering. However, this time can be longer under lower temperatures, as in the study area, or shorter at higher temperatures. Salazar *et al*. [33] reported a duration of 38 weeks from anthesis to fruit ripening for *C. arabica* in the Philippines (14.16^∘^ N and 121.25^∘^ E;), at 39 masl and a mean annual temperature of 27.6 ^∘^C. In another study conducted by Pezzopane *et al*. [34, 35] in Brazil (22^∘^ 54’ S and 47^∘^ 05’ W; 669 masl) on Mundo Novo plantations, the duration from flowering to fruit ripening ranged from 28 to 35 weeks at mean temperatures above 22.5 ^∘^C, while De Oliveira [17] reports, for the cultivar CatuaÃ­-Amarelo-IAC-62, a duration of 24 to 27 weeks from the end of flowering to fruit ripening due to differences in temperature, being longer in areas with low mean temperatures (above 15 ^∘^C, 396 days). The data obtained in the present study during the three productive periods analyzed showed that the duration of growth, fruit filling, and fruit ripening of the coffee plants ranged from 32 to 34 days, very similar to the reports mentioned above. The similarity in the duration of the phenological phases between the periods coincides with similar temperatures between the periods analyzed (average monthly temperature of 18.5 ^∘^C in the three periods); this pattern suggests that temperature plays a central role in regulating the phenological phases of coffee plants.

The water status of coffee plants is closely related to water availability derived from precipitation and the ability of plants to extract water from the soil. In this study, the water status was assessed from plant water demand (ETc/ETo), because this ratio is used for water resource planning and the design of irrigation systems [29]. In the present study, the highest ETc/ETo occurred during the ripening phase for the period 2018–2019 (1.18) and in the flowering phase for the period 2019–2020 (1.24), with mean values of 1.15 and 1.12, respectively.

The mean value of ETc/ETo during the three periods evaluated was 0.97, with the highest value in 2017–2018 and the lowest in 2018–2019, corresponding to the periods with the highest and lowest precipitation, respectively. Under controlled conditions, it has been reported that the maximum water demand of coffee occurs during the flowering and ripening stages, with ETc/ETo values greater than one for the Caturra and Iapar 59 coffee varieties, with mean values of 0.86 to 0.88, respectively [20, 21]. Pereira *et al*. [36] and Oliveira *et al*. [22] reported mean values of 1.04 for Mundo Novo and 0.86 for Arabica and Caturra coffee, respectively.

### Water balance

The study period was characterized by precipitation levels below the monthly averages reported in the climatological averages for the study area. This behavior can be attributed to a reduction in cold fronts (November 2017 to April 2018), in addition to the fact that the crop developed without water stress during the ripening and harvest phases in the period 2017–2018 and during flowering in 2018–2019 period (ETc/ETo > 1). The influence of the La NiÃ±a event intensified these lower rainfall conditions in the study area [37, 38]; however, this lower rainfall contributes to favor positive physiological responses in coffee plants, since flowering and bud break are stimulated [39, 40].

December 2018 was a high-precipitation month (60.3% above average) due to the incidence of cold fronts and low-pressure systems. These conditions were expected to extend throughout the winter season (December-February) due to the influence of the El NiÃ±o event [38]; however, a highly pronounced precipitation deficit (only 23% of the usual precipitation) occurred in January and February 2019. These inter-annual variations in winter precipitation are greatly important not only for coffee cultivation but for agricultural activity in general. Lower rainfall conditions between December and February cause water stress in coffee plants, stimulating flowering and bud break [40, 41]. In contrast, excess water due to high precipitation can cause early and scattered blooms; if rainfall extend to early spring, it could favor the spread of pests and diseases that adversely impact crop yield.

The study area was also affected by low temperatures (<5 ^∘^C) and frosts caused by cold air masses associated with frontal systems. These conditions limited the water demand during the harvesting phase (2018–2019), with a value of 0.63; under favorable conditions for coffee, values close to 1 have been reported. Low temperatures and limited water supply from precipitation inhibit water absorption, inducing stress periods [42]. Under water stress conditions, coffee plants close their stomata, thus reducing gas exchange (transpiration) [43, 44]; in addition, low temperatures (*T*<7 ^∘^C) and water deficit produce chlorosis and inhibit plant growth [32], affecting the duration of fruit ripening and harvest [40, 45].

Between May and October 2017–2018, when the fruit growth and filling phase occurs, coffee plants developed without water stress (ETc/Eto=0.73) because the water demand of the crop was met with the high precipitation levels associated with cyclones, tropical waves, and cold fronts. During the 2018–2019 cycle, the crop experienced low water availability from May to November, caused by the absence of tropical cyclones, reduced activity of tropical waves, and intense intra-summer drought, which is not reflected in the water status of fruit growth and filling (ETc/ETo=0.68); due to the temporal scale of the analysis. In the fruit growth and filling phase of 2019–2020, coffee plants developed without water limitations (ETc/ETo=0.7) despite the lack of tropical cyclones. This water stress value is similar to those reported in coffee systems with chalahuite (*Inga densiflora*) shade (ETc/ETo=0.81) and in monoculture (ETc/ETo=0.61) [28], but is different from the value reported for a controlled system (ETc/ETo=0.49) [20].

### Vegetative development and yield

The growth rate and the number of leaves of coffee plants were positively correlated with precipitation and the amount of water infiltrated into the soil. These represent the main water sources for the crop, available to the plants in the root zone. These results are similar to those reported by Criollo *et al*. [46] and Arcila *et al*. [27], who found a positive relationship between coffee plant growth and precipitation. During dry periods, low soil water availability induces water stress. Since these periods do not occur over a short period of time, plants can develop a range of time-dependent morphological and physiological acclimatization responses [47, 48]. At the morphological level, the mechanisms to conserve water are wilting, leaf rolling, decreased stomatal aperture, loss of succulence, and reduced leaf area and root growth [44, 49, 50].

According to Almeida Silva *et al*. [51], *C. arabica* yields increase with higher Tmin and periods of very low temperatures (December-February) associated with the El NiÃ±o - Southern Oscillation (ENSO) phenomenon. However, Craparo *et al*. [45] reported that the historical increase in Tmin has adversely impacted *C. arabica* yields in Tanzania, while Gay *et al*. [11] reported a similar trend for Mexico. This adverse impact on yield is similar to the one identified with increasing maximum temperatures [52].

Furthermore, according to Silva *et al*. [51] and Avelino *et al*. [53], the interaction of water and thermal variables (Fig 6) can also adversely impact coffee plant yield, as it favors conditions suitable for the incidence and proliferation of coffee diseases such as coffee rust, the most devastating for *C. arabica* [54].

Given the results of the present study, it is important to highlight that the analysis of the hydric variables measured at a greater soil depth would help to better understand the hydric status of the plants. according to Santos *et al*. [55] and Silva *et al*. [56], the hydric conditions between 45 cm and 75 cm depth better reflect the hydric conditions of plants. Furthermore, in the plantation studied, coffee plants were planted in a 0.40 m deep strain where the soil was replaced with compost, a material with a high nutrient content. These nutrients leach with the water that percolates through the soil, promoting the growth of roots into deeper areas that contain nutrients and adequate water conditions [56]. Deep percolation has been considered insignificant in several works; however, its quantification would allow identifying the amount of water that is out of the reach of plant roots [57, 58]. Based on the above, we recommend that studies that analyze the water balance and water status of coffee plants include deep percolation, since it would advance our knowledge of how water is distributed in the root zone and its flow into deeper zones [59, 60].

## Conclusion

The analysis of the data obtained in the present study demonstrates that climatic variability influences vegetative growth, yield, and the water status of coffee plants, and that this effect varies according to the phenological phases of the crop. Our findings indicate that the precipitation recorded at the meteorological station installed at the study site was lower during the periods 2017–2018 (20.1%) and 2018–2019 (14%) compared to the climatological average reported for the region (Briones station, period 1981–2010), this reduction can be attributed to the impact of the El NiÃ±o phenomenon and the lower incidence of cold fronts that occur regularly in the study region. The moisture deficit caused water stress during the ripening and harvesting phases, as evidenced by the findings of the present study. These results indicate that the ripening, harvesting and flowering phases exhibit the highest water demand and are particularly susceptible to water deficits.

During the low-precipitation productive periods (2017–2018 and 2018–2019), we found that the water variables infiltration (I), soil water storage (S), and precipitation (P) led to reduced vegetative growth (growth and number of leaves per plant) of coffee plants. The first and second variables represent the amount of water available to plants in the root zone and depend on the third variable as the main source of water for the crop. Therefore, it is important to conserve a water source during this crop phase to ensure optimal results.

The results of the linear and multiple regression analyses suggest that coffee plants yield is correlated with water and microclimatic variables, since they explained 45% of the variability of yield data. The hydrological variables that best explained yield variability were related to soil water storage (S) and temperature, particularly minimum temperature (Tmin).

These results support the potential benefits of adopting agricultural practices that allow producers to capture and increase rainwater infiltration, such as infiltration drains, planting on contour lines, and improving the structure of coffee plantations by introducing multipurpose trees that provide appropriate shade to the crop and, at the same time, mitigate extreme temperature variations. Undoubtedly, these practices will help coffee growers to mitigate the impact of low-precipitation or drought periods on the development of coffee plants.

## Supporting information

S1 Table Phenological phases and monthly value of maximum temperature and minimum temperature. Behavior of maximum temperature and minimum temperature for each phenological phase in coffee plants.
(XLSX)

S2 Table Phenological phases and monthly value of vegetative growth and number of leaves evaluated in coffee plants located at 1 and 2 m. Vegetative development evaluated at 1 and 2 m distance from the shading tree.
(XLSX)

S3 Table Monthly cumulative precipitation and reference evapotranspiration for each phenological phase in coffee plants. Behavior monthly cumulative precipitation and reference evapotranspiration for each phenoplogical phase in coffee plants.
(XLSX)
